# Robotic-Assisted Total Hip Arthroplasty: Implications for Surgical Practice and Operating Theatre Design

**DOI:** 10.3390/medicina62081428

**Published:** 2026-07-23

**Authors:** Isabelle Middleton, Thomas W. Wainwright, Robert Middleton

**Affiliations:** 1 Independent Researcher, Poole BH12 5BB, UK; 2 Orthopaedic Research Institute, Bournemouth University, Bournemouth BH12 5BB, UK; rmiddleton@bournemouth.ac.uk; 3 Physiotherapy Department, University Hospitals Dorset NHS Foundation Trust, Bournemouth BH7 7DW, UK; 4 Orthopaedic Department, University Hospitals Dorset NHS Foundation Trust, Bournemouth BH7 7DW, UK

**Keywords:** robotic-assisted surgery, total hip arthroplasty, operating theatre design, surgical workflow, human factors, operating room efficiency, patient safety, surgical outcomes, perioperative care

## Abstract

The introduction of robotic-assisted total hip arthroplasty (THA) has had significant effects over and above the more accurate component position it delivers. Diagnosis and templating are improved as a result of the planning CT scan. Complex cases involving abnormal anatomy are simplified surgically by a single ream for the acetabular component and feedback for hip length and offset for the femoral component. The learning curve for surgeons relates primarily to operative time rather than complications. Once planned, robotic systems may reduce variability in component positioning between surgeons with differing levels of experience. Patient Reported Outcome Measures (PROMs) will need to be supplemented with gait lab data and functional tests to prove the added value of robotic-assisted THA. As robotic-assisted surgery (RAS) becomes increasingly reliant on imaging systems and digital workflows, operating theatres face greater complexity in technology integration and intraoperative coordination. Emerging evidence links spatial organisation, equipment positioning, and workflow to operative efficiency, intraoperative safety, and surgical performance. This paper provides an evidence-informed narrative perspective on how advances in robotic-assisted THA are influencing the spatial and functional requirements of operating theatres, thereby affecting clinical performance. As surgical practice advances, there is a need to move towards future-proofed design strategies informed by clinical performance and systems-based evidence. This includes a greater focus on flexibility and integrated digital infrastructure to support evolving technologies, changing team dynamics, and increasing procedural demands. Operating theatres must therefore evolve to enable effective integration of RAS in THA, and support improved patient safety and clinical outcomes.

## 1. Introduction

The development of robotic-assisted surgery (RAS) in total hip arthroplasty (THA) transforms the whole pathway and has significant implications from diagnosis through surgery to outcome assessment. Having a routine CT scan allows not only 3D templating but also assessment of impingement and spinopelvic movement [1]. Where the skill was previously delivering the plan, the plan is now the skill, and RAS delivers it. This may enable surgeons in training and lower-volume surgeons to achieve component positioning approaching that of experienced users.

Recent systematic reviews and meta-analyses have consistently demonstrated that robotic-assisted THA improves the accuracy and reproducibility of acetabular component positioning, leg length restoration, and implant alignment when compared with conventional techniques. Although emerging evidence suggests lower rates of some intraoperative and perioperative complications, as well as reduced revision risk in selected contemporary studies, these radiographic advantages have not yet translated into consistently superior patient-reported outcome measures (PROMs), functional outcomes, or long-term implant survivorship. Consequently, the principal value of robotic-assisted THA currently appears to lie in improving surgical precision and reducing variability in component placement [1,2,3,4].

Although improved implant positioning is biologically and mechanically plausible as a means of enhancing long-term outcomes, contemporary PROMs may lack sufficient sensitivity to detect subtle functional advantages in well-performing hip replacements. Furthermore, modern conventional THA already delivers excellent clinical outcomes for many patients, creating a ceiling effect that makes incremental improvements difficult to demonstrate. Longer-term follow-up and more sensitive objective measures of function may therefore be required to establish whether improvements in surgical precision translate into clinically meaningful patient benefit.

One significant area that hasn’t been widely appreciated is the spatial, technical and functional requirements of modern operating theatres. As theatres become more data-driven, with a growing emphasis on precision, standardisation, and patient-specific outcomes, the operating theatre itself becomes a more significant factor in supporting surgical performance. The integration of robotics, navigation systems, and advanced imaging into routine workflows increases infrastructure complexity and poses risks to operative efficiency, intraoperative safety and surgical outcomes.

Despite these technological advances, most operating theatres remain based on fixed layouts and traditional design principles that were not developed to accommodate such complexity. As a result, current environments are increasingly misaligned with the demands of RAS, particularly in terms of workflow coordination, equipment integration, and spatial organisation. This gap highlights a growing tension between rapidly evolving surgical technologies and current theatre design.

A growing body of empirical research supports the misalignment between technological advancement and the physical environment of the operating theatre. Studies in robotic and orthopaedic surgery demonstrate that operating room layout, size, and infrastructure directly influence workflow efficiency, team coordination, and the frequency of flow disruptions. These environmental factors have been shown to affect staff movement patterns, system setup, and intraoperative communication, with implications for both efficiency and safety [5,6,7].

As robotic platforms continue to advance, and expectations for safety, efficiency, and capacity increase, there is a clear need to move beyond retrofitting existing theatres. Instead, operating theatre design must adopt proactive, future-oriented strategies that anticipate technological change while supporting surgical performance and patient safety. In response to this challenge, this paper examines the impact of operating theatre design on surgical performance, intraoperative safety, and patient outcomes in robotic-assisted THA, using an evidence-informed narrative review informed by a structured, non-systematic search of the contemporary literature.

This review was conducted using an evidence-informed narrative approach rather than a formal systematic review. Literature was identified through searches of PubMed/MEDLINE, Scopus and Google Scholar using combinations of the terms robotic-assisted total hip arthroplasty, robotic surgery, operating theatre, operating room design, operating room workflow, human factors, surgical ergonomics, operating room efficiency, artificial intelligence, and hybrid operating theatre. Reference lists of key articles were also screened to identify additional relevant publications. Priority was given to recent systematic reviews, meta-analyses, high-quality observational studies and influential papers relevant to robotic-assisted orthopaedic surgery, operating theatre design, workflow, human factors and emerging digital technologies. Studies were selected based on their relevance to the themes of this narrative review rather than through predefined systematic review methods.

Although the clinical evidence relating to robotic-assisted THA is well established for topics such as component positioning, surgical accuracy and pre-operative planning, comparatively little research has specifically examined the influence of operating theatre design on robotic-assisted THA. Consequently, where THA-specific evidence is unavailable, this review draws on findings from robotic surgery and orthopaedic operating theatre research more broadly to inform discussion of workflow, human factors and theatre design. These broader findings are interpreted in the context of robotic-assisted THA and identified as such throughout the manuscript.

## 2. Diagnosis

RAS in THA such as the Mako Total Hip 4.0 Robotic-Arm Assisted System, requires a pre-operative CT scan, unlike manual THA, which relies on a plain X-ray. The scan not only supports pre-operative planning but may also identify incidental pathology, which has been in 42% of cases, with 17% requiring further action and 1% showing potential or actual malignancy [8]. The surgeon is therefore aware of multiple pathologies, such as vascular abnormalities, which may affect or prevent surgery, as well as enabling malignancies to be treated at an earlier stage as a result of the CT. Therefore, radiologist reporting of planning CTs is essential and reviewing and acting on the findings is also essential before the patient is admitted for surgery.

These advantages should be considered alongside the potential limitations of CT-based planning, including additional radiation exposure, increased imaging costs, scanner availability and the organisational implications of integrating CT into routine pre-operative pathways. However, modern low-dose CT protocols substantially reduce radiation exposure, and these considerations should be balanced against the potential benefits of improved surgical planning, patient-specific assessment and the identification of clinically important incidental findings.

## 3. Planning in RAS THA

The value of the CT scan in templating is that it allows 3D visualisation of the osteoarthritic hip. Things that are not fully apparent on an X-ray, such as osteophytes can be seen in their exact position, size and orientation. Positioning the components to avoid impingement is possible, along with planning the removal of certain osteophytes. The CT scan is taken from the L2 vertebrae to the joint line of the knees. This allows the surgeon to consider deformity in the spine, pelvis and femur on the ipsi and contralateral leg when planning surgery. Component position can be altered to account for abnormalities of spinopelvic movement [9]. With 3D modelling of the planned THA, the proposed templating can be taken through a range of motion to look for impingement, and alterations can be made to the positioning to improve this.

## 4. Complex Cases Simplified

Planning using three-dimensional CT imaging provides a more complete appreciation of patient-specific anatomy and may therefore facilitate management of complex anatomical deformity. Once the plan for RAS THA is loaded, delivering it to the surgeon is significantly easier. Usually, the issue is in identifying the anatomy in the face of deformity. Identifying anatomical landmarks in the presence of deformity can be challenging. With RAS, the platform guides the surgeon to the exact surgical position. In preparing the acetabulum, robotic guidance may facilitate accurate single-ream preparation even in complex cases.

## 5. Surgical Experience and Outcomes

RAS THA changes the skills required for successful surgery. Before, it was the development of the expert surgeon requiring decades of experience to deliver the plan. Now the skill is in the plan, which can be delivered with more accuracy with RAS than the expert surgeon alone can deliver. Studies suggest that the initial learning curve for robotic-assisted THA is reflected predominantly in operative time rather than increased complication rates. However, the learning process extends beyond technical proficiency and encompasses pre-operative planning, workflow integration, communication, and effective coordination across the multidisciplinary theatre team. As robotic-assisted surgery becomes increasingly embedded within routine practice, successful implementation depends upon both individual technical competence and organisational adaptation. Currently, RAS THA accounts for less than 10% of cases and is used principally by high-volume expert surgeons [10]. As the evidence base evolves, robotic-assisted THA may prove particularly beneficial for surgeons in training and lower-volume surgeons by improving the consistency of component positioning; however, further clinical evidence is required to determine whether these technical advantages translate into improved patient outcomes.

Currently, there is limited evidence of the superiority of RAS THA compared to manual THA despite lower, more accurate component positioning. This is perhaps due to the ceiling effect of current scoring systems. The development of more sensitive outcome assessment tools is required to demonstrate the additional value of robotic-assisted THA [11]. These are likely to include objective functional testing, gait analysis, digital biomarkers derived from wearable technologies, and remote monitoring of recovery trajectories alongside conventional PROMs. Such multidimensional outcome assessment may be better able to detect clinically meaningful differences following technologically advanced surgical interventions.

As robotic-assisted THA increasingly relies on imaging integration, navigation systems, digital workflows, and coordinated intraoperative team interaction, surgical performance becomes increasingly influenced not only by the surgeon but also by the operating theatre environment in which these technologies are deployed.

## 6. Operating Theatre Environment and Its Impact on Surgical Performance

Empirical studies across robotic and orthopaedic surgery have shown that operating theatre layout significantly influences staff movement patterns and clinical workflow efficiency, which affects surgical performance. Much of the current evidence regarding operating theatre design and workflow has been generated from robotic surgery across multiple surgical specialties, with comparatively fewer studies focusing specifically on robotic-assisted THA. Nevertheless, many of the human factors and workflow principles identified are applicable across robotic surgical environments and are likely to have relevance to robotic-assisted orthopaedic procedures. In RAS, poorly configured environments are associated with increased, often avoidable, staff travel, particularly for circulating nurses, as well as congestion in key transition zones [12,13]. These inefficiencies reflect suboptimal spatial organisation, in which misaligned adjacencies among surgical, supply, and support zones increase unnecessary motion and coordination demands.

**Figure 1:**
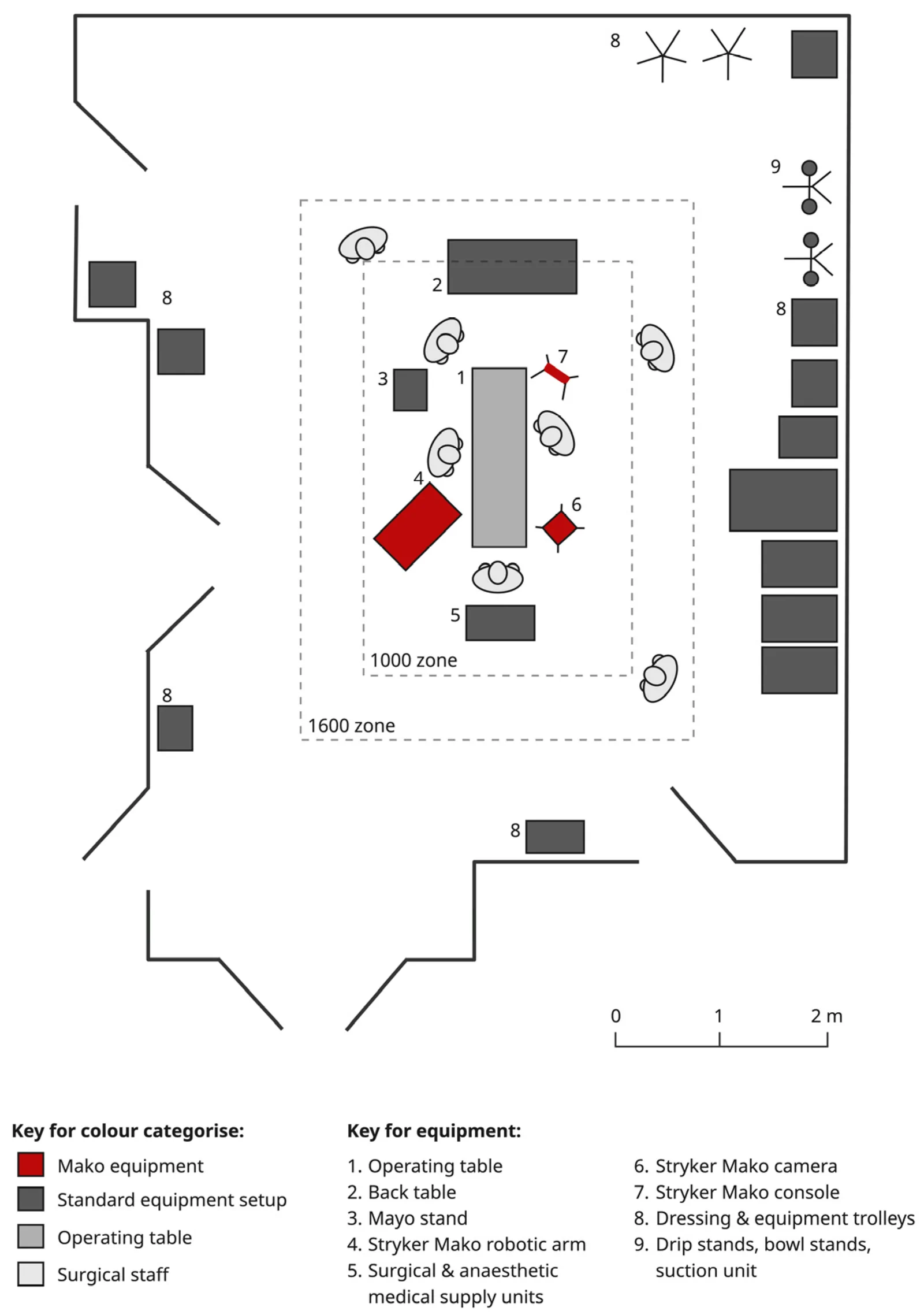
Schematic conceptual floor plan illustrating a typical operating theatre configuration for robotic-assisted total hip arthroplasty (THA), based on current clinical practice and intended to demonstrate representative equipment positioning, staff working zones and circulation pathways rather than a prescriptive or universally applicable layout. The figure highlights the increased spatial requirements and workflow considerations associated with robotic-assisted surgery.

The increasing number of in-theatre technologies further contributes to spatial constraints. Robotic platforms such as the Mako Total Hip 4.0 Robotic-Arm Assisted System (Stryker, Fort Lauderdale, FL, USA) require navigation systems, imaging devices, and display units, increasing the equipment footprint (see Figure 1). Figure 1 illustrates a typical operating theatre configuration for robotic-assisted THA and highlights the increased spatial demands associated with robotic technology. In addition to the operating table and conventional instrument tables, dedicated space is required for the robotic arm, robotic console, navigation equipment and supporting infrastructure. The figure also demonstrates the defined working envelopes surrounding the robotic system, together with the positioning of the anaesthetic team, scrub staff and circulating personnel. These spatial relationships are critical because they influence staff movement, equipment accessibility and communication throughout the procedure. Careful positioning of equipment and clearly defined circulation routes help minimise unnecessary movement, reduce the risk of cable interference and equipment collisions, maintain sterility, and support efficient workflow. Consequently, theatre layout becomes an integral component of successful robotic-assisted surgery rather than simply the physical setting in which the procedure is performed. This can lead to congestion, cable clutter, and repositioning by surgical staff [14,15]. Flow disruptions related to environmental and layout factors are well documented and represent a key mechanism through which theatre design impacts performance. Current evidence suggests that such disruptions are associated with longer operative times and increased risk of error, particularly in complex environments such as RAS [14,15,16]. Collectively, these workflow inefficiencies contribute to increased procedural variability, reduced operative efficiency, and a higher risk of intraoperative complications, with implications for both patient safety and surgical outcomes.

In parallel, spatial interference can also contribute to cognitive and physical workload for surgical staff, as attention shifts between clinical tasks and environmental navigation, affecting concentration, communication, and intraoperative decision-making. Quantitative studies demonstrate the importance of spatial configuration in supporting efficient workflows. Larger operating rooms with improved clearances, optimised equipment positioning, and better access routes have been associated with reduced operative duration, fewer environmental disruptions, and decreased staff movement in orthopaedic procedures [17]. Simulation-based redesign of operating room layouts has also been shown to reduce instrument handover times, minimise staff travel distances, and improve ergonomics [18]. Together, these findings suggest that optimised spatial configuration supports safer, more consistent surgical performance in robotic-assisted and orthopaedic procedures.

The integration of advanced technologies within operating theatres is constrained by limited digital infrastructure and real-time data integration. In many current settings, key systems such as imaging platforms are not fully co-located or integrated with the surgical environment, often requiring patient or equipment transfers between spaces [19]. This separation introduces communication breakdowns and increases coordination demands among surgical teams. Studies show that even small delays or data loss can affect patient safety and disrupt coordination among robots, the surgical team, and supporting infrastructure [20].

At a system level, these inefficiencies have implications for throughput and resource utilisation. Evidence suggests that theatre organisation and design are directly linked to surgical performance, with more efficient environments associated with improved operational outcomes [21]. Given the projected increase in demand for THA, these trends have important implications for healthcare delivery and resource allocation [22]. While robotic systems may offer clinical benefits, their full value in improving clinical workflow and surgical performance is difficult to realise without corresponding improvements in theatre design and workflow integration.

## 7. Emerging and Future Trends in Technology-Driven Surgery

The increasing adoption of robotic-assisted surgery also reflects a broader shift towards healthcare as a complex sociotechnical system, in which clinical performance emerges from interactions between healthcare professionals, digital technologies, organisational processes and the physical environment. Within this framework, successful implementation depends not only on technological capability but also on the adaptability of teams, effective communication, workflow integration and organisational resilience. Consequently, operating theatre design should be viewed as supporting the performance of the wider surgical system rather than simply accommodating new equipment.

Emerging developments in robotics have led to smaller, more compact devices, as demonstrated by systems such as Think Surgical, a miniature, handheld, wireless robotic platform with an overhead active-tracking device for total knee arthroplasty (TKA). Such systems enable improved patient access with a minimal robotic bedside footprint. Furthermore, evidence suggests that AI technologies enable real-time decision support, predictive analytics, and semi-autonomous actions in the operating theatre [23]. Together, these developments reflect a shift in surgical practice toward greater integration of robotics and AI-enabled systems.

The development of “smart” or context-aware theatres enables real-time data exchange, automated documentation, and improved coordination across surgical teams and departments. This involves integrating active tracking systems, such as Real-Time Location Systems (RTLS), Radio Frequency Identification and optical sensors into the operating theatre. These sensor-based tracking technologies and real-time monitoring systems provide detailed insights into staff movement, equipment utilisation, and workflow bottlenecks [13]. Evidence from literature reviews suggests that hybrid theatres, compared with conventional theatres, have higher procedural accuracy, reduced operative time, and reduced haemorrhage during transport between radiology departments and operating suites [24].

Operating theatres should also adapt to changing procedural and technological requirements. Theatre design should be flexible enough to integrate emerging technologies, rather than requiring those technologies to be adapted to outdated spaces [25]. In orthopaedic surgery, optimised layouts reduce procedure duration, improve ergonomics, and decrease unnecessary movement through better positioning of equipment and personnel [17,18]. This includes clearly defined zoning for robotic systems, imaging equipment, and staff movement pathways. Improved layout design has the potential to reduce unnecessary movement, minimise workflow disruptions, and support operative efficiency and workflow reliability.

The widespread adoption of robotic-assisted surgery will also depend upon economic sustainability. Although robotic systems require substantial capital investment, their value proposition increasingly includes potential improvements in surgical accuracy, workflow efficiency, operating theatre utilisation and long-term healthcare outcomes. Future evaluations should therefore consider not only clinical effectiveness but also cost-effectiveness, procedural volume, service configuration and the centralisation of complex robotic services.

## 8. Towards Evidence-Based Operating Theatre Design

While there is a growing body of empirical evidence demonstrating the impact of operating theatre design on clinical workflow and surgical performance, the translation of this evidence into clinical practice and practical theatre design guidance remains limited. In particular, prospective studies evaluating how operating theatre configuration influences workflow efficiency, surgical performance and patient outcomes specifically during robotic-assisted THA remain scarce. This represents an important area for future research as adoption of robotic-assisted orthopaedic surgery continues to expand. Systematic reviews and observational studies consistently show that layout, room size, and infrastructure influence surgical duration, staff movement, and coordination [5,21,26]. Moving towards evidence-based design requires a more integrated approach that considers the interactions among technology, clinical teams, workflow performance and the built environment. In addition, future research should prioritise adaptable design strategies that can accommodate ongoing technological change, rather than relying on retrospective modifications to existing spaces. This will help ensure that next-generation operating theatres are not only technologically advanced but also responsive to evolving surgical demands.

## 9. Conclusions

The development of RAS in THA is changing the pathway from diagnosis and pre-operative planning through to surgical execution and outcome assessment. The routine use of CT-based planning enables more detailed assessment of anatomy, spinopelvic movement, impingement, and incidental pathology, while robotic systems allow increasingly accurate and reproducible delivery of the surgical plan. These advances may be particularly beneficial in complex cases and for surgeons in training or lower-volume practice.

As robotic-assisted THA increasingly relies on imaging integration, navigation systems, and coordinated digital workflows, the operating theatre environment itself becomes more important for supporting surgical performance and patient safety. However, most current operating theatres remain designed around the requirements of conventional THA procedures and are not optimised to accommodate the spatial, technological, and workflow demands introduced by robotic-assisted surgery. This misalignment may contribute to workflow inefficiencies, increased cognitive and physical workload, intraoperative disruption, and reduced operative efficiency.

Current evidence suggests that improvements in workflow efficiency and reductions in intraoperative disruptions are associated with safer and more reliable surgical care. By reducing operative duration, minimising unnecessary movement, and improving team coordination, optimised environments may indirectly contribute to improved clinical outcomes [17,21,26]. As demand for THA continues to rise, alongside increasing technology complexity, there is a need to move beyond retrofitted solutions towards operating theatre environments designed specifically to support robotic-assisted and technology-driven surgical care.

Ultimately, the successful integration of RAS depends not only on advances in technology but also on the environments in which they are deployed. Developing evidence-based, future-proof operating theatres that support workflow efficiency, intraoperative safety, and clinical performance will be essential to maximising patient safety and high-quality surgical performance. Looking ahead, operating theatres are likely to evolve into increasingly connected digital environments in which robotics, artificial intelligence, interoperable information systems and real-time clinical data support integrated surgical decision-making. Designing operating theatres that facilitate both physical workflow and digital connectivity will be fundamental to realising the full potential of technology-enabled orthopaedic surgery.

## Data Availability

No new data were created or analyzed in this study.

## Abbreviations

The following abbreviations are used in this manuscript:

RAS	Robotic-assisted surgery
THA	Total hip arthroplasty
TKA	Total knee arthroplasty
RTLS	Real-Time Location Systems
